# Preliminary study on the influencing factors of Young’s modulus of viscoelastic model detected by shear wave elasticity

**DOI:** 10.3389/fmed.2025.1684745

**Published:** 2025-12-02

**Authors:** Aifen Wu, Maosheng Xu, Shijia Wang, Xiu Chen, Yi Zhou, Bin Zhou, Chunpeng Zou, Yanhua Huang, Ying Li

**Affiliations:** 1 Department of Ultrasound Medicine, Lishui Municipal Central Hospital, The Fifth Affiliated Hospital of Wenzhou Medical University, Lishui, Zhejiang, China; 2 Department of Ultrasound Imaging, The Second Affiliated Hospital of Wenzhou Medical University, Wenzhou, Zhejiang, China; 3 Department of Ultrasound Medicine, The First Affiliated Hospital of Ningbo University, Ningbo, Zhejiang, China; 4 Department of Thyroid and Breast Surgery, Lishui Municipal Central Hospital, The Fifth Affiliated Hospital of Wenzhou Medical University, Lishui, Zhejiang, China

**Keywords:** shear wave elasticity, viscoelastic model, viscosity, influencing factor, ultrasonic frequency

## Abstract

**Aim:**

The objective of this study is to investigate the influence of various components, viscosity, ultrasonic frequency, and depth of the region of interest on Young’s modulus of the viscoelastic model.

**Methods:**

Viscoelastic models, characterized by distinct viscosities and compositions, were fabricated using gelatin as the elastomeric component, fructose as the viscous component, and milk powder as an additive ingredient. Shear wave elastography (SWE) technology was utilized, with the acoustic frequencies set at 7 MHz, 8 MHz, and 9 MHz, respectively. Additionally, the depths of regions of interest (ROI) were varied at 1 cm, 2 cm, and 3 cm to enable a comparison of Young’s modulus values across different viscoelastic body models.

**Results:**

In order to generate viscoelastic models with varying compositions, gelatin samples with the same concentration were prepared with different concentrations of fructose added. Under consistent frequency and ROI depth conditions, no statistically significant differences were observed in the measured Young’s modulus values among these models (*p* > 0.05). However, for models composed solely of gelatin, significant differences in Young’s modulus values were noted at varying sound wave frequencies and ROI depths (*p* < 0.05). When samples with different fructose and powdered milk compositions and concentrations were prepared at a constant gelatin concentration, a range of viscoelastic models with diverse properties was created. Analysis of the Young’s modulus values at varying sound wave frequencies and ROI depths revealed a statistically significant difference for all model groups when the frequency was 8 MHz and the ROI depth was 3 cm. Moreover, using linear regression analysis on these models after the addition of powdered milk showed that frequency had a significantly negative impact on the Young’s modulus, with a regression coefficient of −0.488 (*t* = −7.341, *p* < 0.01), while depth demonstrated a significantly positive influence on the shear modulus, with a regression coefficient of 0.480 (*t* = 7.158, *p* < 0.01).

**Conclusion:**

The self-constructed viscoelastic model demonstrates convenience and adaptability, allowing flexible control over its viscosity and composition. Under varying conditions, the factors determining Young’s modulus differ; elements such as compositional heterogeneity, frequencies, and ROI depth all influence the modulus.

## Introduction

Shear wave elastography (SWE) is an advanced ultrasound-based imaging technique that enables the quantitative assessment of tissue stiffness (1, 2). The underlying principle of SWE is based on the direct relationship between the propagation speed of shear waves (V) in tissue and the tissue’s shear modulus (*μ*). For a purely elastic, isotropic, and incompressible medium, this relationship is defined by μ = ρV², where ρ is the tissue density. Under the common assumption of tissue incompressibility, the shear modulus can be converted to Young's modulus (E), which is more frequently used in clinical practice (3, 4). Generally, the stiffer the tissue, the faster the shear waves travel (5). By tracking the propagation of shear waves, SWE can quantitatively calculate the tissue’s Young’s modulus. This contrasts sharply with earlier traditional elastography techniques, such as strain elastography, which provide only qualitative or semi-quantitative information. Traditional methods rely on pressure applied by the operator, making the results susceptible to operator technique and difficult to compare across different examinations. The technical advantage of SWE lies in its objective quantification, offering unprecedented, reproducible biological markers for clinical diagnosis, disease staging, and treatment monitoring. This capability has greatly expanded its clinical applications in areas such as liver fibrosis (6), characterization of breast and thyroid nodules (7, 8), tumor treatment evaluation (9), and various musculoskeletal injuries and pathological conditions (10).

However, the practical application of SWE is influenced by various factors, and its reliability, repeatability, and the factors affecting it remain controversial (11). First, equipment and technical parameters include ultrasound excitation frequency, measurement depth, the static pressure applied by the probe, as well as the size and location of the region of interest (ROI) (12, 13). Acoustic waves at different frequencies interact differently with tissues, which may affect the excitation and detection efficiency of shear waves (14); increased depth can lead to a decline in signal quality. Additionally, the biophysical properties of the tissue being measured—including tissue viscoelasticity, anisotropy (such as the orientation of muscle fibers), heterogeneity, and mechanical constraints from surrounding tissues—significantly influence shear wave propagation, thereby affecting the measurement of Young’s modulus (15, 16).

Therefore, conducting phantom studies on SWE technology has become a crucial step in validating its performance and elucidating various influencing factors. Phantoms, as standardized models with known mechanical properties, enable the systematic investigation of these individual variables (17, 18). A recent study comprehensively investigated the viscoelastic characterization of models using shear wave propagation and also confirmed the value of shear wave methods for model characterization (19). A large number of studies have been conducted to quantify Young’s modulus using SWE, such as assessing the stiffness of individual muscles, idiopathic granulomatous mastitis (IGM), and the appearance of liver tissue lesions (20–22). These studies have identified that both technical parameters and tissue characteristics are critical. However, a significant gap remains. Most existing research treats these influencing factors in isolation, failing to capture their potential interactions. For example, it is still unclear how the effect of measurement depth varies in materials with different viscosity levels, or whether the impact of ultrasound frequency is modulated by heterogeneity in the medium’s composition. This lack of systematic, multifactorial analysis limits our ability to predict and correct the measurement discrepancies that arise when these factors coexist and interact in complex real-world clinical scenarios.

To fill this gap, our study goes beyond validation on a single device or an isolated analysis of a single variable. We utilized custom viscoelastic phantoms to design a comprehensive multifactorial experiment. Our unique contribution lies in systematically quantifying the interactive effects of two key technical parameters (acoustic wave frequency, ROI depth) and two key material properties (viscosity, compositional heterogeneity) on the Young’s modulus derived from SWE. Our ultimate goals are to: (i) identify specific combinations of parameters and material properties where measurement variability is most pronounced and (ii) provide quantitative experimental evidence to explain observed differences between various SWE systems and examination protocols. This approach offers a more detailed and integrated understanding of factors interfering with SWE measurements, representing a critical step toward developing robust correction algorithms and standardized clinical guidelines to improve diagnostic consistency.

## Materials and methods

### Materials

#### Preparation of a viscoelastic model

**Figure 1:**
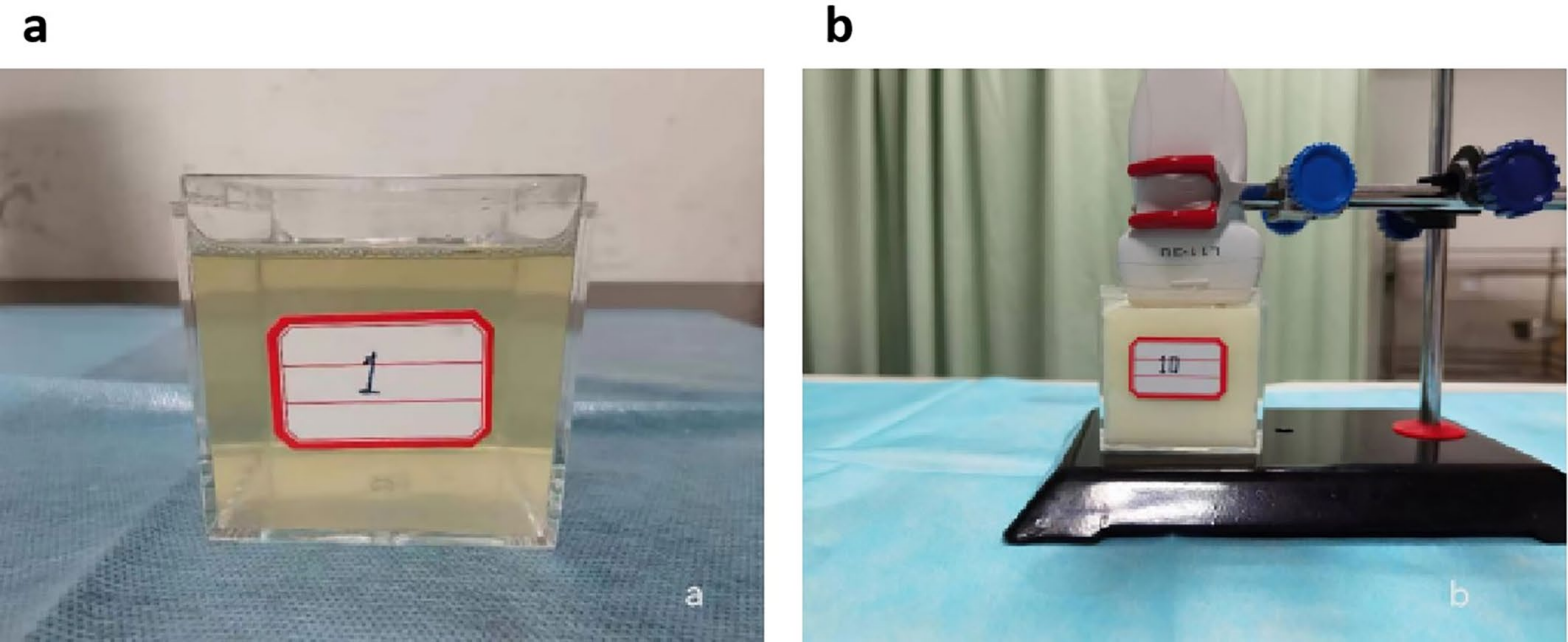
Completed viscoelastic body model. **(a)** Homogeneous, light yellow, transparent viscoelastic model containing 5% gelatin. **(b)** Shows a white, uniform viscoelastic model containing 5% gelatin, 20% fructose, and 20% milk powder.

Preparation steps: ① 10 pieces of solid gelatin were weighed at 15 g and placed in beakers, numbered 1–10, respectively. ② A measure of 20 g of distilled water was poured into the No. 1–10 beakers were immersed, and the solid gelatin was pre-soaked for 15 min, to avoid the gelatin particles from clumping during the production process. ③ Distilled water was added in the following amounts to the molten gelatin mixtures in beakers No. 1–10: 265 g, 235 g, 205 g, 175 g, 145 g, 125 g, 235 g, 205 g, 205 g, and 145 g, respectively, and stir thoroughly until completely dissolved. ④ Weighed amounts of 30 g, 60 g, 90 g, 120 g, 150 g, 30 g, and 60 g of fructose were added to mixtures No. 2, 3, 4, 5, 6, 9, and 10, respectively, and stirred thoroughly until completely dissolved, 2 to 6 mixed solution fructose concentration of 10, 20, 30, 40, 50%; The fructose concentrations of No. 9 and No. 10 mixtures were 10 and 20%, respectively. ⑤ A measure of 30 g, 60 g, 30 g, and 60 g of milk powder was weighed and added to mixtures 7, 8, 9, and 10, respectively, and stirred thoroughly until completely dissolved. The milk powder concentrations in mixtures 7 to 10 are 10%, 20%, 10%, and 20%, respectively. A series of mixtures containing the same gelatin concentration but varying fructose and milk powder concentrations was thus prepared (Table 1). ⑥ All models were cooled and cured at room temperature. ⑦ Before the experiment, the room temperature was measured with a room thermometer, and the reading was 23 ± 2 °C (Figure 1).

**Table 1:** Quality and concentration table of each component of the viscoelastic mixture.

Quality of each component (g)				Concentration of each component (%)			
Number	Gelatin	Fructose	Milk powder	Distilled water	Gelatin concentration	Fructose concentration	Milk powder concentration
1	15	0	0	285	5	0	0
2	15	30	0	255	5	10	0
3	15	60	0	225	5	20	0
4	15	90	0	195	5	30	0
5	15	120	0	165	5	40	0
6	15	150	0	145	5	50	0
7	15	0	30	255	5	0	10
8	15	0	60	225	5	0	20
9	15	30	30	225	5	10	10
10	15	60	60	165	5	20	20

### Viscosity measurement of the viscoelastic body model

A digital rotary viscometer (SNB-4, Shanghai Yixin Scientific Instrument Co., Ltd.) is used to measure viscosity. A measure of 25 mL of the prepared viscoelastic model mixture was poured into the test container, and the temperature of the mixture was measured to be 38 °C. The 0# rotor was selected to connect to the instrument and inserted into the liquid until it was completely immersed. To test the viscosity of the mixture, start the power supply of the viscosimeter, select the appropriate speed, and press the “confirm” button. The viscosity of the mixture (unit: mPa·s) is shown on the display screen at the end of the measurement, with a confidence interval of 10–90% and a percentile. Each model mixture was continuously measured three times, and the average value was calculated (Table 2).

**Table 2:** Viscosity table of each viscoelastic mixture (unit: mPa·s).

Number	1	2	3	4	5	6	7	8	9	10
Viscosity	5.69	6.83	9.34	14.12	19.92	40.24	9.79	20.49	10.07	23.68

### Instruments and methods

#### Elastography of the viscoelastic body model

##### Instrument

**Figure 2:**
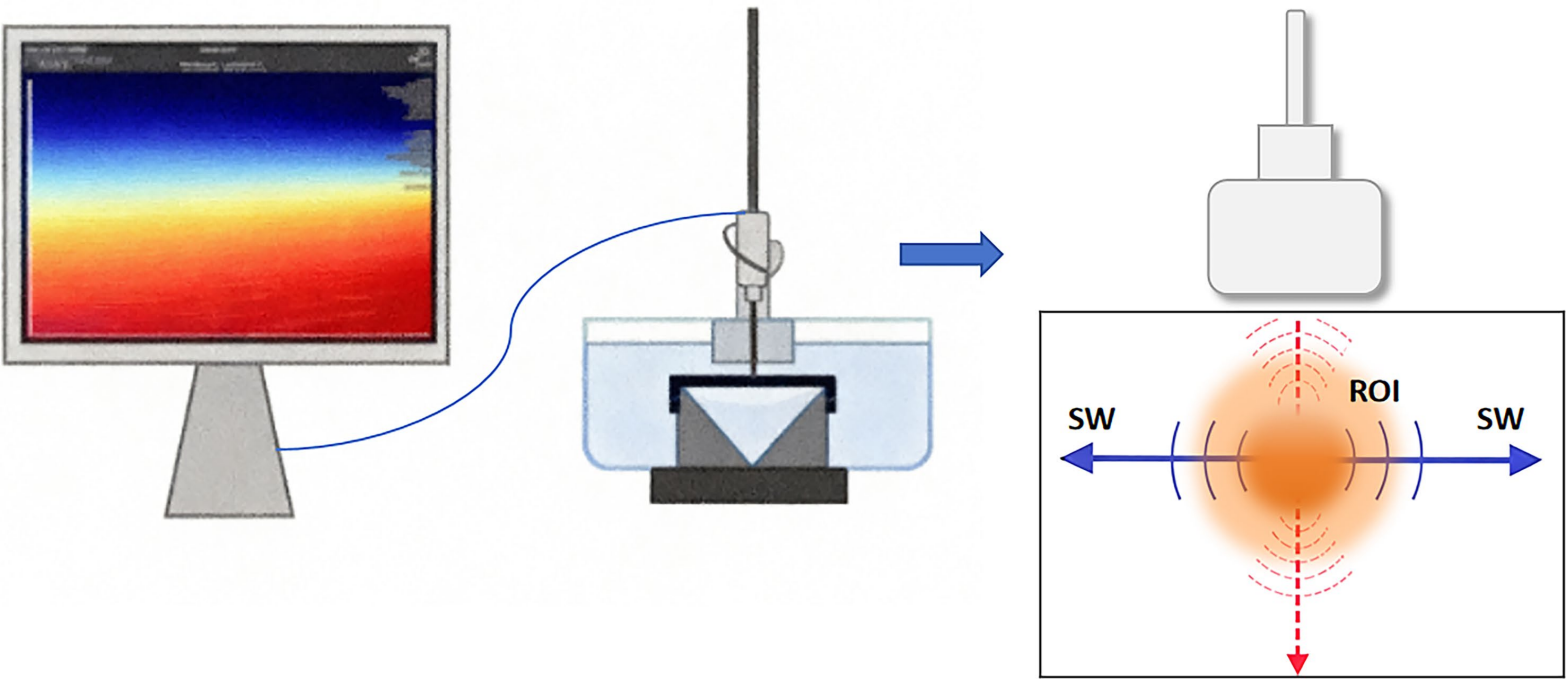
Principles of shear wave elastography. Ultrasound waves are focused on generating shear waves near a region of interest (ROI). The results are as follows: A: 10.34 ± 1.44; B: 11.70 ± 1.14; C: 12.37 ± 0.93, (x̅ ± s), kPa.

The Mindray Resona 7 T color Doppler ultrasound diagnostic instrument was used, with an L11-3 U linear array probe and a frequency range of 3–11 MHz. It also has virtual touch shear wave elastography (STE). The probe was fixed on the experimental scaffold, and its surface was completely in contact with the ultrasonic viscoelastic body model. The probe mode was selected for the superficial organ, and each parameter was adjusted to make the image display clearer before entering STE mode. Acoustic output was 93.33%; the thermal index in soft tissue (TIS) was 0.8; and the diameter of the region of interest (ROI) was set to 5 mm (Figure 2). The sampling site should avoid the uneven area displayed in the elastic image. The images were collected, and the Young’s modulus value (unit: kPa) was recorded. The operator was a doctor who had received professional training in shear wave elastography, mastered relevant knowledge, had more than 5 years’ experience in ultrasound examination, and had completed more than 50 cases of elastography independently. This study uses shear wave elastography to assess the stiffness of the model. The measurement results are expressed as Young’s modulus (E), which the device automatically calculates based on the assumption that the tissue is incompressible (Poisson’s ratio ν=0.5). The calculation uses the measured shear wave velocity (Cs) according to the formula E = 3*ρ*Cs^2^, where ρ represents the density, assumed to be 1,000 kg/m^3^.

#### Criteria for successful measurement of Young’s modulus value

① The motion stability index (M-STE index) ≥ 4 stars is shown in green (≤3 stars are shown in red, indicating poor stability). Red indicates a large range of displacement fluctuation in the current region, and the stability of elastography is significantly affected by displacement. Below three stars, the displacement amplitude in the region of interest > 3 mm/s. ② The region of interest should avoid the red and yellow-green regions and instead choose the blue region.

#### Measurement of Young’s modulus value

**Figure 3:**
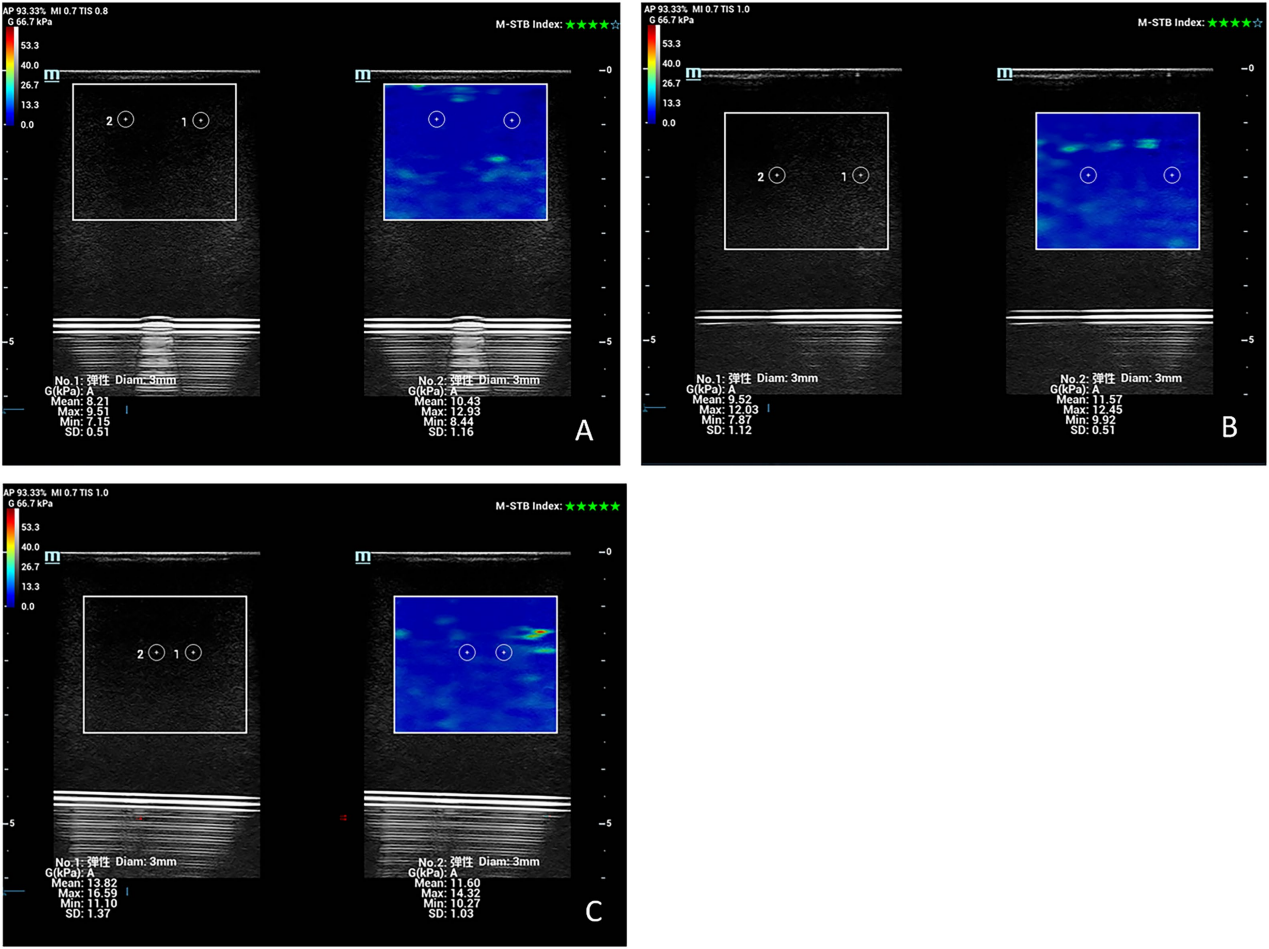
Shear wave elastography images of a set of samples of the No. 10 viscoelastic model are depicted at a probe frequency of 7 MHz and region of interest (ROI) depths of 1 cm **(A)**, 2 cm **(B)**, and 3 cm **(C)**.

The Young’s modulus values of different regions of interest were measured by changing the depth of the sampling frames with different ultrasonic frequencies. Six separate samples were prepared for each experimental condition. Each sample was measured three times, and the average of these measurements was used as a representative value for subsequent statistical analysis. ① The Young’s modulus value of the ROI depths of 1 cm, 2 cm, and 3 cm was measured using an ultrasonic frequency of 7 MHz using a linear array probe. The above operation was repeated with an ultrasonic frequency of 8 MHz and 9 MHz. ② The above methods were used to measure the viscoelastic body models of No. 1–10 (Figure 3).

### Statistical methods

SPSS 25.0 statistical software was used for data statistical analysis. All measurement data were expressed as mean ± standard deviation (x ± s), and count data were expressed as frequency. Graphs and data were used to show the experimental results. The Kolmogorov–Smirnov test was used for the normality of measurement data. One-way ANOVA was used to compare the Young’s modulus among multiple groups. SNK test was used for pairwise comparison when the variance was equal, and the Dunnett T3 test was used for pairwise comparison when the variance was not equal. The independent influencing factors of Young’s modulus were analyzed by linear regression analysis. A *p*-value of <0.05 was considered statistically significant.

## Results

### Appearance and physical characteristics of the ultrasonic viscoelastic body model

**Figure 4:**
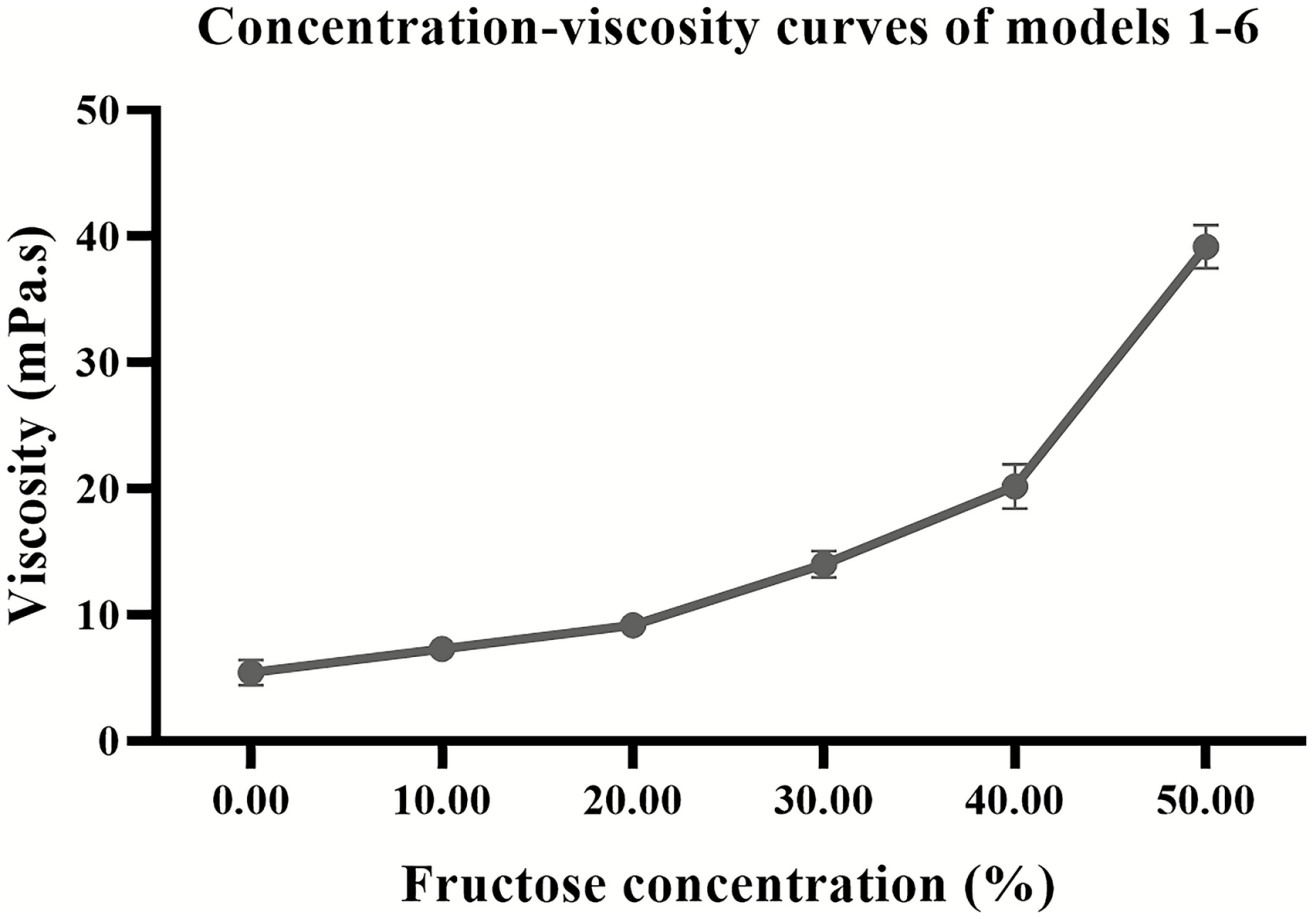
1–6 fructose concentration–viscosity curves of the viscoelastic model.

The viscoelastic model was cuboid in shape, with a uniform and elastic texture. Models 1–6 had a pale yellow and transparent overall appearance, while models 7–10 appeared to be white. The viscosity values of models 1 to 6 indicated that as the fructose concentration increased, the viscosity of the mixture also increased (Figure 4).

### Comparison of Young’s modulus values of viscoelastic body models with different fructose concentrations at different frequencies and depths

**Figure 5:**
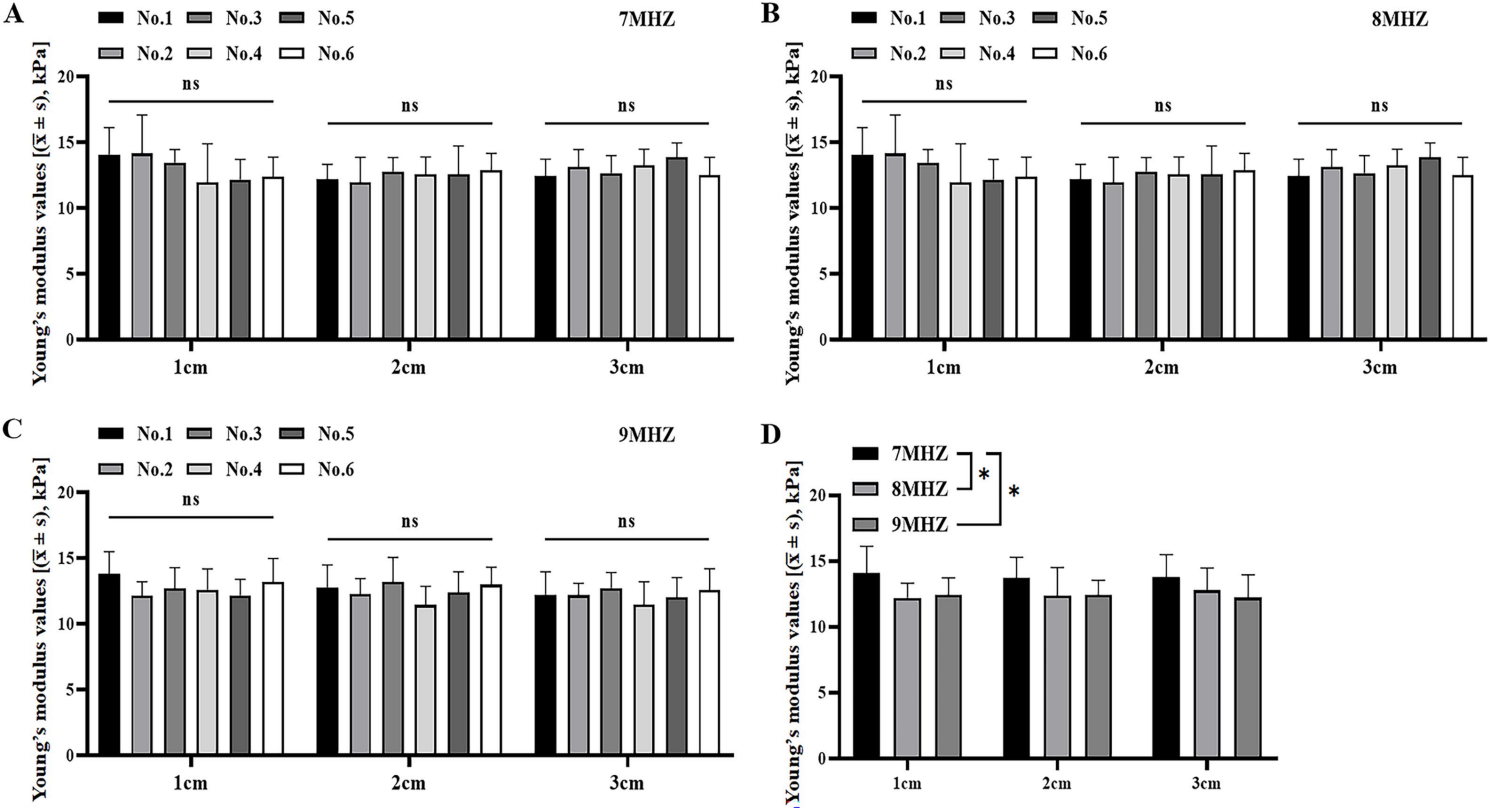
Comparison of Young’s modulus values of viscoelastic body models with varying fructose concentrations and viscosities at different frequencies and depths. **(A)** Comparison of Young’s modulus values of different viscoelastic models at 7 MHz frequency and various depths. **(B)** Comparison of Young’s modulus values of different viscoelastic models at 8 MHz frequency and various depths. **(C)** Comparison of Young’s modulus values of different viscoelastic models at 9 MHz frequency and various depths. **(D)** Comparison of Young’s modulus values in Model 1 under different frequencies and depths. *p < 0.05.

There was no significant difference in the Young’s modulus values of each model under the same frequency and the same ROI depth in No. 1–6 viscoelastic body models (*p* > 0.05) (Table 3; Figures 5A–C). However, significant differences in the Young’s modulus values were observed between different frequencies and depths within model 1 (*p* < 0.05) (Table 3; Figure 5D). There was no significant difference in the Young’s modulus values between models 2–6 (Table 3).

**Table 3:** Comparison of Young’s modulus values of viscoelastic body models with different fructose concentrations and viscosities at different frequencies and depths.

Frequency and depth		Young’s modulus values [(x̅ ± s), kPa]						*F*	*p*
		No. 1	No. 2	No. 3	No. 4	No. 5	No. 6		
7MHZ	1 cm	14.08 ± 2.04	14.16 ± 2.92	13.46 ± 0.99	11.95 ± 2.95	12.16 ± 1.55	12.38 ± 1.50	0.303	0.951
	2 cm	12.20 ± 1.12	11.94 ± 1.92	12.77 ± 1.07	12.58 ± 1.31	12.57 ± 2.16	12.88 ± 1.28	0.332	0.910
	3 cm	12.42 ± 1.30	13.14 ± 1.32	12.65 ± 1.34	13.25 ± 1.22	13.87 ± 1.09	12.50 ± 1.36	0.711	0.747
8MHZ	1 cm	13.70 ± 1.59	13.29 ± 1.58	12.84 ± 0.77	11.84 ± 1.55	13.21 ± 1.89	12.85 ± 1.74	1.371	0.359
	2 cm	12.36 ± 2.15	13.14 ± 1.64	12.02 ± 1.34	12.59 ± 1.48	12.66 ± 1.00	13.45 ± 1.62	1.410	0.595
	3 cm	12.43 ± 1.11	12.69 ± 1.59	12.88 ± 1.43	12.16 ± 0.94	12.39 ± 1.54	12.89 ± 1.53	0.866	0.655
9MHZ	1 cm	13.79 ± 1.70	12.14 ± 1.07	12.69 ± 1.24	12.59 ± 1.59	12.15 ± 1.24	13.18 ± 1.80	0.579	0.835
	2 cm	12.78 ± 1.70	12.26 ± 1.18	13.19 ± 1.87	11.44 ± 1.42	12.37 ± 1.58	12.98 ± 1.33	1.013	0.391
	3 cm	12.23 ± 1.73	12.18 ± 0.91	12.72 ± 1.19	11.45 ± 1.76	12.00 ± 1.53	12.58 ± 1.61	4.450	0.121
*F*		4.714	2.206	1.217	0.034	0.837	0.670		
*p*		0.0138^*^	0.114	0.299	0.966	0.435	0.513		

### Comparison of Young’s modulus values of viscoelastic bodies with different compositions and concentrations at different frequencies and depths

**Figure 6:**
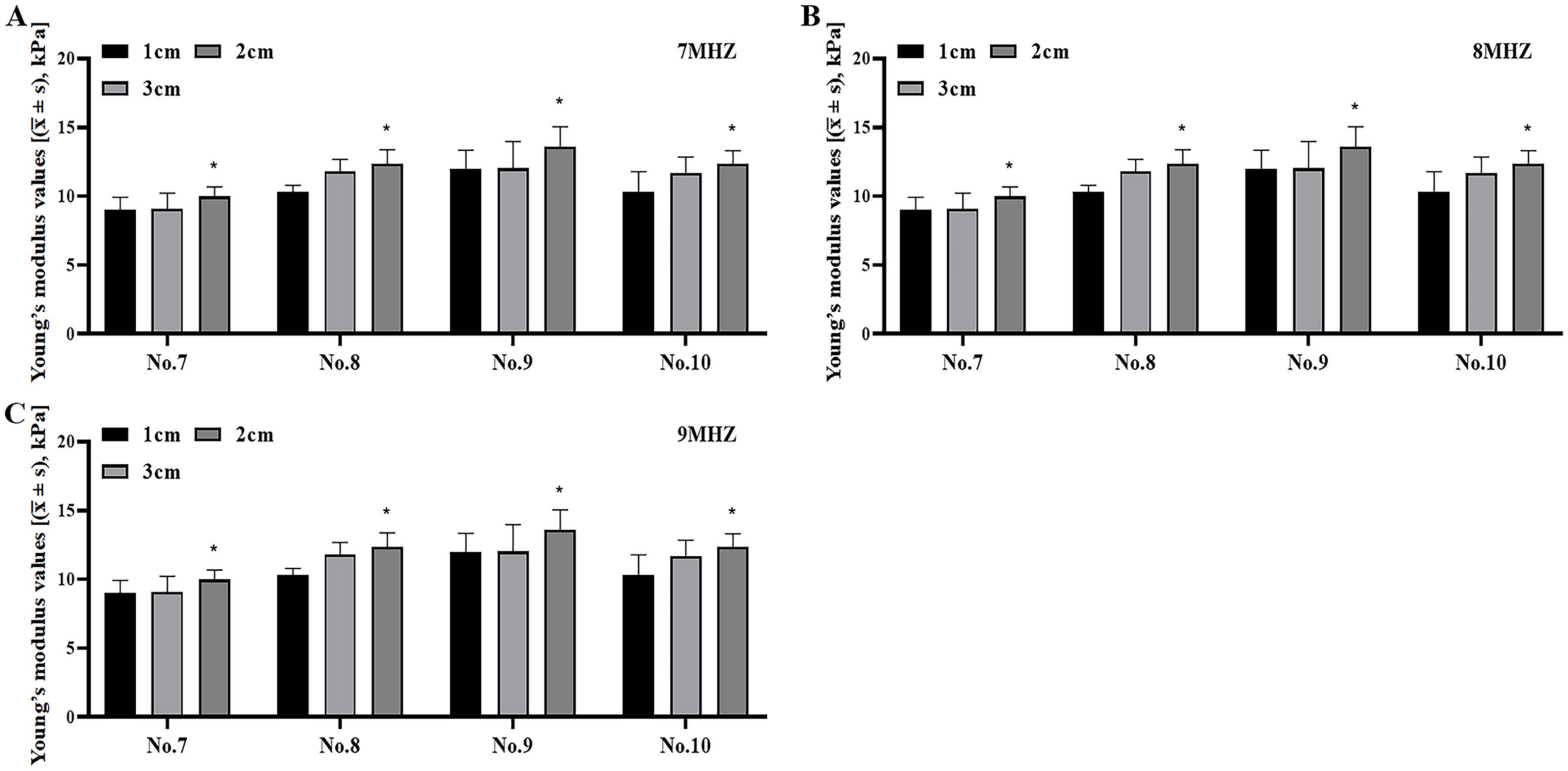
Compare the Young’s modulus values of No. 7–10 samples at the same frequency and at different depths. **(A)** Comparison of Young’s modulus values at 7 MHz frequency and at different depths. **(B)** Comparison of Young’s modulus values at 8 MHz frequency and at different depths. **(C)** Comparison of Young’s modulus values at 9 MHz frequency and at different depths. *p < 0.05 vs. 1 cm.

**Figure 7:**
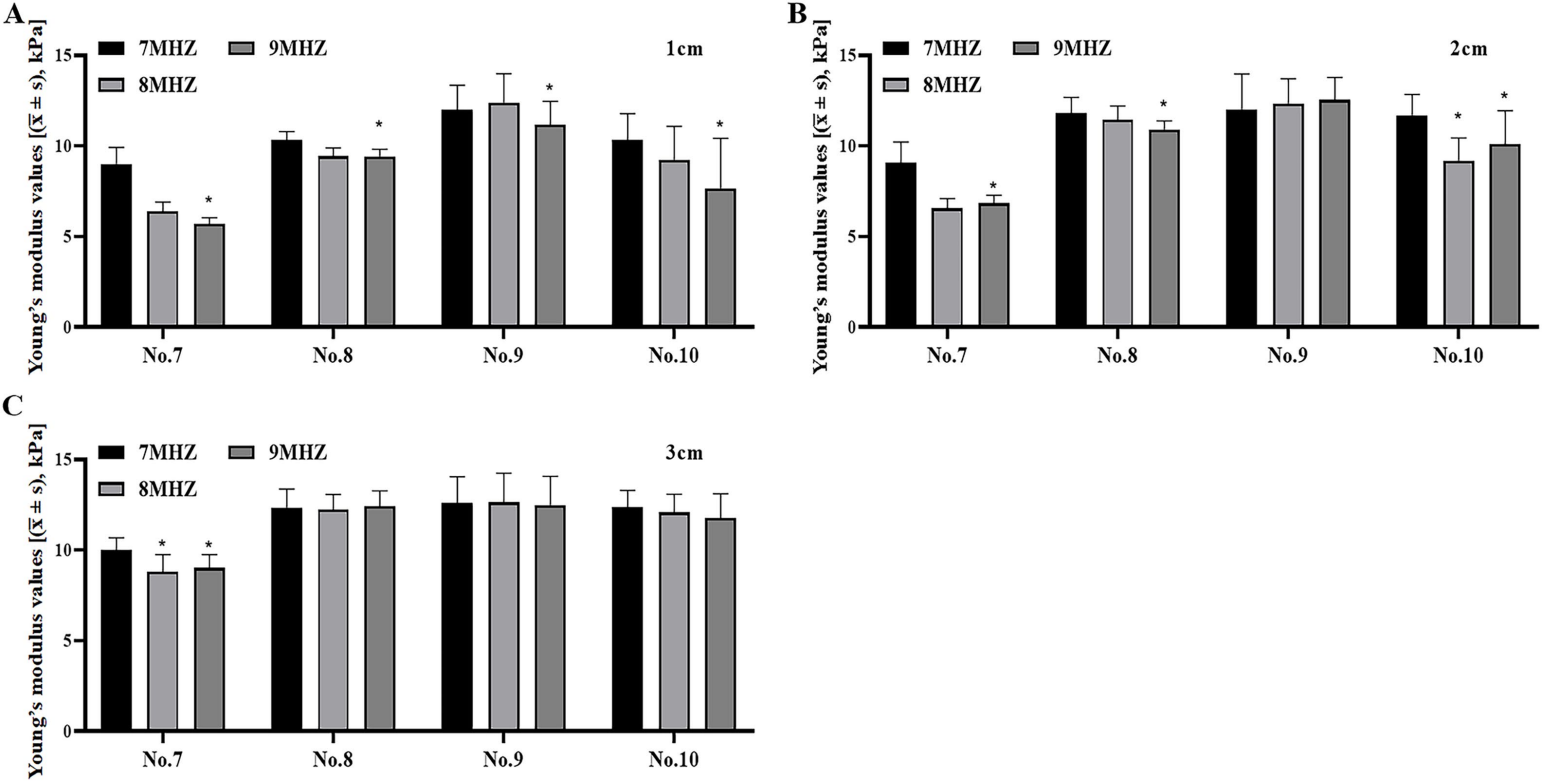
Values of Young’s modulus of No. 7–10 samples at different frequencies at the same depth were compared. **(A)** Comparison of Young’s modulus values at 1 cm and different frequencies. **(B)** Comparison of Young’s modulus values at 2 cm and different frequencies. **(C)** Comparison of Young’s modulus values at 3 cm and different frequencies. *p < 0.05 vs. 7MHZ.

There were significant differences in the measurement of Young’s modulus values between different frequencies and ROI depths in No. 7–10 models (*p* < 0.05) (Table 4). To investigate the relationship between Young’s modulus and the frequency and depth of viscoelastomer models with varying components and viscosities, we compared the Young’s modulus values of samples No. 7–10 at different frequencies and depths. The results showed that the Young’s modulus value increased with the increase of depth (Figures 6A–C), while the Young’s modulus value decreased after the increase of frequency (Figures 7A–C).

**Table 4:** Comparison of Young’s modulus values of viscoelastic body models with different composition and viscosity values at different frequencies and depths.

Frequency and depth		Young’s modulus values [(x̅ ± s), kPa]						*F*	*p*
		No. 2	No. 3	No. 7	No. 8	No. 9	No. 10		
7MHZ	1 cm	14.16 ± 2.92	13.46 ± 0.99	9.01 ± 0.91	10.32 ± 0.47	12.01 ± 1.33	10.34 ± 1.44	3.005	0.0282
	2 cm	11.94 ± 1.92	12.77 ± 1.07	9.08 ± 1.14	11.81 ± 0.87	12.02 ± 1.95	11.70 ± 1.14	1.365	0.604
	3 cm	13.14 ± 1.32	12.65 ± 1.34	9.99 ± 0.69	12.34 ± 1.03	12.61 ± 1.43	12.37 ± 0.93	0.857	0.665
8MHZ	1 cm	13.29 ± 1.58	12.84 ± 0.77	6.39 ± 0.52	9.43 ± 0.46	12.39 ± 1.59	9.24 ± 1.84	1.256	0.622
	2 cm	13.14 ± 1.64	12.02 ± 1.34	6.57 ± 0.58	11.44 ± 0.77	12.34 ± 1.37	9.20 ± 1.24	1.459	0.587
	3 cm	12.69 ± 1.59	12.88 ± 1.43	8.80 ± 0.95	12.23 ± 0.84	12.63 ± 1.61	12.08 ± 1.00	6.785	0.02
9MHZ	1 cm	12.14 ± 1.07	12.69 ± 1.24	5.69 ± 0.35	9.42 ± 0.40	11.15 ± 1.30	7.67 ± 2.75	4.295	0.127
	2 cm	12.26 ± 2.18	13.19 ± 1.87	6.85 ± 0.43	10.91 ± 0.47	12.54 ± 1.24	10.11 ± 1.84	1.194	0.469
	3 cm	12.18 ± 0.91	12.72 ± 1.19	9.02 ± 0.74	12.41 ± 0.86	12.48 ± 1.59	11.78 ± 1.33	1.239	0.358
*F*		2.206	1.217	58.771	154.816	3.069	51.804		
*p*		0.114	0.299	6.04E-20^*^	5.47E-41^*^	0.048^*^	2.16E-19^*^		

Through correlation analysis, it is found that the correlation value between the Young’s modulus value and the frequency and depth of the viscoelastic body model with different components and viscosities is significant, and the correlation value between the Young’s modulus value and the frequency is −0.171. The correlation value between Young’s modulus and depth was 0.168 (Table 5). The value of Young’s modulus was negatively correlated with frequency and positively correlated with depth. The frequency had a significant negative effect on the Young’s modulus value, and the frequency regression coefficient value was −0.488 (*t* = −7.341, *p* = 0.000 < 0.01). The depth had a significant positive effect on the Young’s modulus value, and the regression coefficient of depth was 0.480 (*t* = 7.158, *p* = 0.000 < 0.01) (Table 6).

**Table 5:** Correlation analysis and comparison of influencing factors of viscoelastic body models with different components and concentrations.

Variables	Mean value	Standard deviation	Shear modulus	Viscosity	Frequency	Depth
Shear modulus	11.650	2.251	1			
Viscosity	16.459	9.358	0.030	1		
Frequency	8.013	0.824	−0.171**	0.055*	1	
Depth	2.051	0.815	0.168**	0.011	0.034	1

* *p* < 0.05; ** *p* < 0.01.

**Table 6:** Regression analysis of influencing factors of viscoelastic body models with different components and concentrations.

Variables	Regression coefficient	95% CI	VIF
Constant	14.426** (26.162)	13.345 ~ 15.507	-
Viscosity	0.009 (1.553)	−0.002 ~ 0.021	1.003
Frequency	−0.488** (−7.341)	−0.618 ~ −0.358	1.004
Depth	0.480** (7.158)	0.349 ~ 0.611	1.001
Sample size	324		
*R* ^2^	0.061		
Adjust *R*^2^	0.059		
*F* value	34.425***		

** *p* < 0.01; *** *p* < 0.001.

## Discussion

Shear wave elastography (SWE), the latest non-invasive technology for quantitatively assessing tissue stiffness, has been widely adopted in clinical practice. The fundamental imaging principle of SWE is based on an ideal elastic body that is uniform, linear, isotropic, and infinitely extensible in the plane. However, human tissues and organs possess complex structures and mechanical properties that can affect the practical application of SWE. Several studies have demonstrated that the measured values and variability of SWE are influenced by system factors such as the region of interest (ROI) depth and the average frequency of shear wave vibration (23). Consequently, shear wave elasticity values obtained under different conditions often vary. A thorough understanding and explanation of the reasons behind these differences, as well as an investigation into the potential factors influencing SWE results, is crucial for improving the accuracy of tissue and organ elasticity measurements. This, in turn, will enhance the clinical utility of SWE in disease diagnosis, severity assessment, and the development of precise treatment plans.

In this study, the viscosity of the mixed liquid was measured at a constant room temperature using a viscometer to minimize measurement errors caused by temperature fluctuations. Gelatin served as the matrix, and fructose was added at varying concentrations to simulate a homogeneous medium with different viscosities. The measurements indicated that as the fructose concentration increased, the viscosity also increased, consistent with the findings of Gong et al. (24). Gelatin’s mechanical properties closely resemble those of biological soft tissue, making it an ideal material for fabricating viscoelastic body models. By incorporating different components and varying their concentrations within the gelatin matrix, viscoelastic body models with distinct viscosity gradients can be prepared, allowing flexible control over the model’s composition and shape (25).

When propagating through a viscoelastic medium, the speed of shear waves increases with frequency, indicating that dispersion occurs during the propagation process (26, 27). Liver tissue exhibits both elastic and viscous properties. Clinically, conditions such as acute hepatitis, abnormal liver function, and biliary obstruction can increase the liver’s viscosity component, leading to measurement errors (28). Huang et al. (29) created spherical nodule phantoms with varying viscosities and embedded them into background phantoms of different hardness and depths. Their results showed that the elastic modulus of the nodule phantoms increased with increasing density and viscosity. In this study, when the model was composed of two components—gelatin and fructose—the Young’s modulus measurements were not affected by viscosity under consistent frequency and region of interest (ROI) depth conditions. Analysis revealed that the viscosity of the model ranged from 5.69 mPa·s to 40.24 mPa·s, whereas Huang Yunlin et al. reported viscosities ranging from 465.00 mPa·s to 1290.00 mPa·s in their nodule phantoms, which is significantly higher. The authors speculate that there exists a specific viscosity range within which shear wave elastography values correlate with viscosity (30). In Huang Yunlin et al.’s model, structures with different components and viscoelastic properties existed between the nodule and background phantoms. The liver, as a medium, has a more complex structure and composition, exhibiting component heterogeneity during shear wave measurements, as well as spatial variations within the viscoelastic body (2). Furthermore, this study found significant differences in Young’s modulus values measured at different frequencies in model 1 (*p* < 0.05). This may be related to the fact that model 1 is a pure gelatin model. Compared to the model containing fructose, the pure gelatin model has different components, resulting in variations in shear wave displacement frequency and attenuation along the propagation path. Some studies (31) have found that, compared to oil-free gelatin models of the same concentration, shear waves propagating through gelatin with castor oil generate higher-frequency shear wave displacements and exhibit less attenuation along the propagation path. Additionally, gelatin exhibits non-linear characteristics, and the addition of castor oil can effectively reduce both the hardness and non-linear behavior of the model, while making the internal structure more uniform and stable. Therefore, the authors speculate that the uneven spatial distribution within the pure gelatin model may be due to insufficient dissolution during the production process. The results of this study indicate that the Young’s modulus values of the viscoelastic model with varying fructose concentrations are not influenced by frequency or depth. Potthof et al. reported in a prospective study that the reproducibility of ARFI elastography in assessing liver stiffness varies with measurement depth. Lu Chang et al. (32) used sound touch elastography (STE) to examine different regions of the bilateral renal parenchyma in 68 healthy volunteers and found that STE results were unaffected by the distance between the middle capsule of the left kidney and the probe, as well as by sampling depth (33). This discrepancy may be attributed to differences in detection outcomes among various elastography technologies and manufacturers. Numerous elastography techniques are used in clinical practice, each with distinct diagnostic criteria, normal ranges, and potential influencing factors depending on the instrument.

Since the structure of human tissues and organs is complex and variable, uniform, single-component models cannot adequately capture this complexity. In this study, the effect of various factors on the measured Young’s modulus was further investigated by adding milk powder to the model to simulate the heterogeneity of the medium. The results suggest that when the region of interest (ROI) depth is held constant, the Young’s modulus decreases as the frequency increases. Conversely, when frequency is held constant, the Young’s modulus increases with increasing ROI depth. These changes may be related to variations in the components of the viscoelastic model. Several studies have indicated that elastic heterogeneity is a key characteristic distinguishing diseased tissues from normal tissues (25, 33–36). Elastic heterogeneity refers to the varying stiffness within different regions of a lesion due to spatial and histological heterogeneity, which arises from changes in the regional mechanical properties caused by alterations in the lesion’s internal components (37). Additionally, one study found that the presence of fatty liver significantly reduced the ability of SWE to differentiate liver fibrosis stages. It was hypothesized that the increased accumulation of cellular lipid droplets in the liver alters the homogeneity of the medium, thereby affecting shear wave propagation (38). The results of this study demonstrated that SWE values of viscoelastic models with varying components and viscosities differed across ultrasound frequencies and ROI depths. These findings suggest that when using SWE to measure tissue stiffness clinically, the heterogeneity of tissue composition should be taken into account, and the results should be interpreted in conjunction with other underlying tissue pathologies to obtain more objective and reliable assessments.

The limitations of this study are as follows: ① The range of viscosity values for each component in this study was narrow, the number of models was limited, and the sample size was small. ② The components of the ultrasound viscoelastic body model developed in this study were relatively few, and no model was created to simulate lesions. There is no comparison between human tissues. Additionally, there was no comparison with human tissue. The structure and composition of the human body are complex and variable, influenced not only by the pulsation of large blood vessels but also by the differing physical properties of its components in various regions. ③ It was observed that the viscosity of the viscoelastic model affected Young’s modulus differently under varying conditions; however, the specific viscosity range that influences shear wave elasticity measurements remains unclear, necessitating further experiments. ④ In this study, the variation between probe frequency and the depth of the region of interest during Young’s modulus measurement was minimal, and additional experiments are required to explore this further.

## Conclusion

The self-made viscoelastic model can flexibly control the viscosity and composition. Under the condition of different components, the influencing factors of Young’s modulus are different. Component heterogeneity, ultrasound frequency, and ROI depth are all influencing factors of SWE.

## Data Availability

The original contributions presented in the study are included in the article/supplementary material, further inquiries can be directed to the corresponding author.
